# Racial Bias in Perceptions of Others’ Pain

**DOI:** 10.1371/journal.pone.0048546

**Published:** 2012-11-14

**Authors:** Sophie Trawalter, Kelly M. Hoffman, Adam Waytz

**Affiliations:** 1 Frank Batten School of Leadership and Public Policy, University of Virginia, Charlottesville, Virginia, United States of America; 2 Department of Psychology, University of Virginia, Charlottesville, Virginia, United States of America; 3 Kellogg School of Management, Northwestern University, Evanston, Illinois, United States of America; The University of Hong Kong, Hong Kong

## Abstract

The present work provides evidence that people assume *a priori* that Blacks feel less pain than do Whites. It also demonstrates that this bias is rooted in perceptions of status and the privilege (or hardship) status confers, not race *per se.* Archival data from the National Football League injury reports reveal that, relative to injured White players, injured Black players are deemed more likely to play in a subsequent game, possibly because people assume they feel less pain. Experiments 1–4 show that White and Black Americans–including registered nurses and nursing students–assume that Black people feel less pain than do White people. Finally, Experiments 5 and 6 provide evidence that this bias is rooted in perceptions of status, not race *per se*. Taken together, these data have important implications for understanding race-related biases and healthcare disparities.

## Introduction

Relative to White Americans, Black Americans experience higher rates of diseases, disability and premature death [1], [2]. Disparities in healthcare contribute to these health disparities. Black patients are more likely to receive lower-quality healthcare and are subject to less desirable procedures. For instance, Black patients are over three times more likely than White patients to have limbs amputated as a result of diabetes [3]. Moreover, Black patients are systematically undertreated for pain [4]–[6]. They are less likely than Whites to receive pain medication and, when they do, they receive less [7], [8]. Numerous explanations have been proposed, ranging from assumptions about Black patients’ inability to pay for healthcare to racial prejudice [6], [9]. These explanations generally imply that Black patients’ pain is recognized but not treated. Another explanation, however, is that Black patients pain is not recognized in the first place. The present work begins to examine this possibility; it provides evidence that people–including medical personnel–assume *a priori* that Black people feel less pain than do White people.

Consistent with this thesis, a study of physician-patient interactions has shown that physicians underestimate Black patients’ pain more than White patients’ pain [10]. Because this study was not an experiment, however, it is not clear whether this bias was the result of patient race, physician characteristics, and/or characteristics of the patient-physician interaction. Social psychological research provides relevant but inconclusive experimental evidence for our thesis. Work on stereotyping and prejudice has shown that Blacks, Black men in particular, are stereotyped as being dangerous and physically tough–qualities that might make them seem impervious to pain [11]–[14]. Work on dehumanization has shown that Black men are infra-humanized and that the infra-humanization of Black men is associated with the condoning of police brutality against Black men [15]. These findings suggest that people do not care about harm inflicted upon a Black victim and/or that they do not recognize the extent to which a Black victim might be injured by such harm. Finally, work on the “intergroup empathy gap” has shown that Whites often fail to “feel” the pain of outgroup members, including Black people [16], [17]. Studies using fMRI technology have shown that for White participants, a network of neural regions involved in processing one’s own pain (“the pain matrix”) responds similarly to viewing harm inflicted on racial ingroup but not racial outgroup members [18], [19]. Again, these findings suggest that people do not care about Blacks’ pain and/or do not recognize how much pain Blacks might feel. In the present work, we tested the latter possibility. We provide experimental evidence that people, including medical personnel, assume *a priori* that Blacks feel less pain than do Whites. We also provide archival evidence to illustrate the potential breadth of this phenomenon.

### Archival Study

We began testing our hypothesis using the National Football League’s (NFL) 2010 and 2011 injury reports. Throughout the football season, coaching staffs and team medical personnel must evaluate injured players and rate their likelihood of being able to play the following week. We reasoned that if Black players are assumed to feel less pain, then they might be rated as more likely to play when injured relative to White players.

### Methods

Research assistants blind to study hypotheses transcribed the NFL Injury Reports for the 2010 and 2011 seasons. Research assistants recorded each injury for each season, the players’ race, age, experience (years) in the NFL, position, and injury type, as well as players’ next-game status. Next-game status ranged from Out (definitely not playing) to Doubtful, Questionable, and Probable. This ordinal classification system served as our dependent measure.

### Results and Discussion

We constructed a multi-level model to examine the effect of player race on next-game status. We did this for all injuries aside from concussions and illnesses. See Table 1 for a list of injuries. Control variables all affected next-game status; position: *F* (21, 5530) = 1.91, *p* = .008, injury: *F* (56, 5530) = 3.49, *p*<.0001, and experience: *F* (1, 474) = 3.82, *p* = .05. As predicted, the analysis revealed that relative to injured White players, injured Black players were deemed more likely to play in the next game, controlling for players’ experience in the NFL, position, and injury type, *F* (1, 5530) = 6.39, *p* = .01, *M_Black_* = 1.97, *SE_Black_* = .11, and *M_White_* = 1.84, *SE_White_* = .12. We also examined Huber-White standard errors allowing for heteroscedasticity clustered at the team, team-year, and player level. These standard errors are larger than classical standard errors but similar to each other. Results held when we used these conservatively large standard errors in our inferences. Results also held when taking out data from Tom Brady and/or the Patriots; Tom Brady of the Patriots was placed on the injury list almost every week despite playing every (or nearly every) game. Interestingly, players’ race had no effect on next-game status in the case of concussions and/or unspecified illnesses, *F*<1.

**Table 1:** List of injuries for football seasons 2010 and 2011.

Injury	Frequency	Percent
Knee	1803	22.78
Ankle	1255	15.86
Hamstring	783	9.89
Shoulder	683	8.63
Foot	463	5.85
Groin	417	5.27
Back	339	4.28
Calf	228	2.88
Hip	185	2.34
Toe	179	2.26
Neck	167	2.11
Quadriceps	145	1.83
Thigh	133	1.68
Head	131	1.66
Ribs	116	1.47
Elbow	112	1.42
Hand	95	1.2
Thumb	90	1.14
Wrist	89	1.12
Chest	65	0.82
Finger	64	0.81
Shin	46	0.58
Forearm	45	0.57
Abdomen	38	0.48
Fibula	38	0.48
Rib	32	0.4
Achilles	29	0.37
Biceps	23	0.29
Triceps	23	0.29
Pectoral	13	0.16
Pelvis	12	0.15
Glutes	10	0.13
Heel	10	0.13
Eye	9	0.11
Oblique	6	0.08
Migraine	5	0.06
Arm	4	0.05
Lower Leg	4	0.05
Stinger	4	0.05
Arch	3	0.04
Jaw	2	0.03
Kidney	2	0.03
Leg	2	0.03
Back Spasm	1	0.01
Cheek	1	0.01
Collar bone	1	0.01
Dehydrated	1	0.01
Ear	1	0.01
Eye Lid	1	0.01
Hernia	1	0.01
Infection	1	0.01
Lacerated Kidney	1	0.01
Nose	1	0.01
Tibia	1	0.01
Tooth	1	0.01

These findings are consistent with our claim that Black people–in this case, Black players–are presumed to feel less pain than White people. Indeed, it is telling that when mandated standardized testing (rather than human judgment) was used to determine a player’s next-game status, as is the case with concussions, the racial bias disappeared. Although a racial difference emerged among NFL players, these data are far from conclusive. Assuming that Black players feel less pain is one of many reasons why injured Black players might be more likely to play compared with injured White players. For example, it is possible that Black players are more likely to want to play when injured or that they have been socialized to ignore and play through their pain [12].

## Experiment 1

Although these NFL injury data are provocative, the effect of race was small and alternative explanations abound (e.g., players’ determination to play even while injured). We thus sought more direct and conclusive evidence for our hypothesis by conducting a set of experiments. In our first experiment, we tested whether Whites assume that Black people feel less pain than do White people.

### Methods

#### Ethics statement

All studies were approved by the Institutional Review Board at the University of Virginia and conducted in the U.S. All participants provided consent, either by signing a written consent form or indicating their consent by clicking on a button on an online (written) consent form.

#### Participants

We recruited 250 White participants from the University of Virginia (UVA) Department of Psychology participant pool (*N* = 102) and via Mechanical Turk (*N* = 148), an online marketplace powered by Amazon.com. UVA participants received course credit for their participation. Mechanical Turk participants received $0.50 for their participation. We excluded 10 participants from our analyses below for not being native English-speakers and/or American. In all experiments, we excluded non-English-speakers and non-Americans because we suspect this racial bias in pain perception is a cultural phenomenon. Including these participants in our analyses does not change the results of Experiments 1, 2, 4, 5, and 6 but does change the results of Experiment 3 (see below). The final sample of 240 varied in age (*M* = 28.47, *SD* = 12.16) and gender (63% female).

#### Stimuli

We used standardized pictures from the Productive Aging Lab Face Database [20]. Specifically, we used 9 pictures each of Black and White men, and 6 pictures each of Black and White women. Pilot testing revealed that Black and White, male and female targets were rated as equally attractive, emotionally expressive, and familiar, all *Fs*<1. However, the female targets and White targets were rated as significantly less threatening than the male targets and Black targets, respectively, *F* = 21.81, *p*<.0001 and *F* = 5.11, *p* = .03. These differences were expected. They reflect commonly held stereotypes about gender and race, beliefs about what it means to be male or female, Black or White. Indeed, we acknowledge the possibility that perceived threat is part of this racial bias in pain perception and address this potential “threat” confound in Experiment 4.

#### Procedure

After signing (or clicking “continue” to indicate agreement with) the consent form, participants were asked to rate the amount of pain they would feel in 18 situations. Situations ranged from getting a paper cut and getting shampoo in the eye, to getting an injection in the arm, stubbing a toe on a chair, and slamming a hand in a car door. Then, participants were randomly assigned to rate the amount of pain a Black or White gender-matched target person would feel in the same 18 situations. A subset of female participants (*N* = 63) saw a male target; i.e., not a gender-matched target. Excluding these participants does not change the pattern of results. Participants made all of their ratings on 4-point scales (1-*not painful*, 2-*slightly painful*, 3-*moderately painful*, 4-*extremely painful*). This pain measure for self and other was internally reliable, α = .85. Next, participants completed measures of race-related attitudes and/or concerns (i.e., the *Motivation to Respond without Prejudice Scale* [21]; the *White Guilt Scale* [22]; the *Modern Racism Scale* [23]; the *Implicit Association Test* [24]). Finally, participants were asked a number of demographic questions including age, gender, race/ethnicity, social economic status (education/parental education, household income, and subjective social class), nationality (country of birth), and number of years in the U.S.

### Results and Discussion

**Figure 1:**
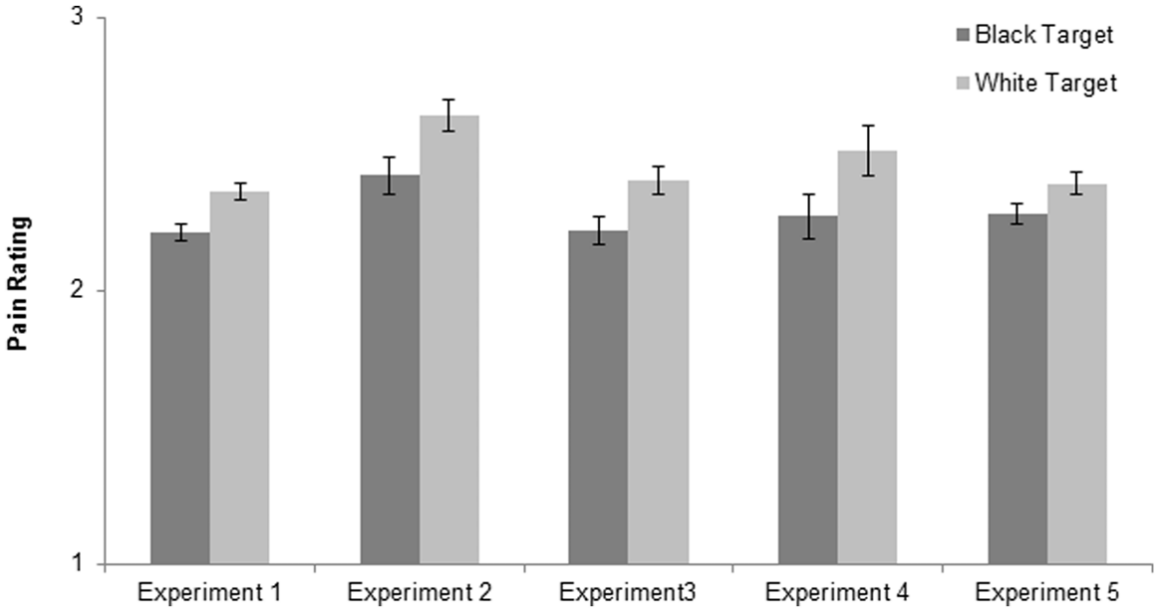
Pain ratings (estimated means and standard errors) for Experiments 1–5.

We constructed a general linear model (GLM) to examine the effect of target race on perceptions of pain. We controlled for participants’ age, gender, and self-ratings of pain. We controlled for age because all targets were young adults, making them more similar to younger participants. Indeed, across experiments, age was often a significant predictor of participants’ ratings of the target’s pain. We controlled for gender given our *a priori* assumption that women would report more pain, both for themselves and for the target. Finally, we controlled for self-ratings of pain because these self-ratings were so variable (with some participants reporting relatively low levels of pain across scenarios and others reporting relatively high levels of pain across scenarios) and so highly predictive of participants’ ratings of the target’s pain. Across experiments, the best predictor of pain ratings was self-ratings of pain. See Table 2 for test statistics for all covariates, for all experiments. More importantly, and consistent with predictions, participants’ pain ratings were significantly lower for a Black vs. White target, *F*(1,235) = 15.07, *p* = .0001, *d* = .51. See Figure 1. This result held when not controlling for covariates. As a brief aside, the effect of target race on pain ratings also held in Experiment 5 but not in Experiments 2, 3, 4, and 6–experiments in which cell sizes are relatively smaller. Our sense is that self-ratings of pain are too variable and too predictive to be ignored. The interested reader can look at the Supporting Information for tables of unadjusted means and standard deviations for self-ratings of pain and ratings of the target’s pain, and correlations between self-ratings of pain and ratings of others’ pain.

Explicit and/or implicit race-related attitudes and/or concerns did not moderate this effect, suggesting that this bias in pain perception is not the result of racial prejudice *per se*. In other words, although we observed a difference in the way people treated (perceived) a Black vs. a White target (i.e., a racial bias in pain perception), this difference was not associated with negative or otherwise demeaning thoughts, feelings, or action tendencies toward Black people more generally (i.e., racial prejudice).

**Table 2:** Test statistics for covariates in experiments 1–5.

	Experiment 1	Experiment 2	Experiment 3	Experiment 4	Experiment 5	Experiment 6
Self-ratings	*F* = 123.74	*F* = 61.40	*F = *37.41	*F* = 54.79	*F* = 130.40	*F* = 366.53
	*p*<.0001	*p*<.0001	*p*<.0001	*p*<.0001	*p*<.0001	*p*<.0001
Age	*F* = 3.57	*F* = 7.78	*F*<1	*F* = 5.24	*F*<1	*F* = 2.99
	*p* = .06	*p* = .01		*p* = .03		*p* = .08
Gender	*F*<1	*F*<1	*F* = 1.40		*F* = 3.67	*F*<1
			*p* = .24		*p* = .06	
Race/ethnicity			*F*<1	*F*<1	*F*<1	*F*<1

## Experiment 2

The fact that racial bias in perception of others’ pain was not related to explicit or implicit race-related attitudes and/or concerns raises the possibility that this bias is not rooted in racial animus, at least not primarily or entirely. Thus, in Experiment 2, we replicated Experiment 1 with Black participants, reasoning that Black Americans might also show the bias.

### Methods

#### Participants

We recruited 42 Black participants from the UVA Psychology participant pool (*N* = 17) and via Mechanical Turk (*N* = 25). UVA participants received course credit for their participation. Mechanical Turk participants received $0.50 for their participation. We excluded 7 participants from the analyses below for not being native English-speakers and/or American. Including these participants in our analyses does not change the pattern of results below. Our final sample of 35 varied in age (*M* = 30.22, *SD* = 14.08) and gender (67% female).

#### Procedure

The procedure was identical to that of Experiment 2 with the exception that participants did not complete any of the race-related measures.

### Results and Discussion

We constructed another GLM to examine the effect of target race on pain ratings, again controlling for participants’ age, gender, and self-ratings of pain. Analyses revealed a similar bias. Participants’ ratings were significantly lower for a Black vs. White target, *F* (1,30) = 5.27, *p* = .03, *d* = .84. See Figure 1. These findings suggest that this bias is not rooted solely in racial prejudice or intergroup dynamics.

## Experiment 3

In our introduction, we claim that this bias may shed light on racial disparities in healthcare and, specifically, pain treatment. To begin to investigate this claim, we replicated Experiments 1 and 2 with a sample of registered nurses and nursing students.

### Methods

#### Participants

We recruited 56 participants with the help of faculty members and administrators at a school of nursing. Participants were mailed a $10 gift certificate for their participation. Thirteen identified the main hypothesis, and thus their data were removed. It is worth noting that most of these participants completed the study toward the end of data collection, suggesting that they had heard about the study from someone else. Including these participants in our analyses did change the results–the pattern did not change but the difference between target race conditions was no longer statistically significant. The final sample of 43 included 29 registered nurses and 14 nursing students. The sample varied in age (*M* = 32.64, *SD* = 12.84) and ethnicity (88% White, 7% Black, and 5% other). All participants except one were women.

#### Procedure

The procedure was identical to that of Experiment 2.

### Results and Discussion

We constructed a GLM to examine the effect of target race on ratings of pain, controlling for participants’ age and self-ratings of pain. We did not control for gender as only one participant was male. However, we controlled for participant race given that we had participants from various ethnic/racial groups. We reasoned, based on Experiments 1 and 2, that there might be ethnic/racial group differences in pain ratings; namely, that Black participants might systematically report greater pain than White participants (see Supporting Information). As in the first two experiments, participants’ pain ratings were significantly lower for a Black vs. White target, *F* (1,38) = 4.90, *p* = .03, *d* = .72. See Figure 1. In other words, nurses and nursing students in this study also assumed that Blacks feel less pain than do Whites.

## Experiment 4

Experiments 1–3 provide some support for our thesis that people–including nurses and nursing students–assume *a priori* that Blacks feel less pain than do Whites. Recall, however, that independent coders rated the Black targets as significantly more threatening than the White targets (Experiment 1). It is thus possible that participants assumed that threatening individuals feel less pain than do non-threatening individuals; not that Blacks feel less pain than do Whites. This explanation of our data is not quite satisfactory, however. Extant research has demonstrated that individuals often *over*-perceive threat in Black targets [25], [26]. In this way, perceived threat is not a confound. Being perceived as a threat is part of what it means to be Black in America [27]. Indeed, we suspect that perceived threat might be part of our effect. Nonetheless, we wanted to rule out the possibility that perceived threat was a confound in our stimuli. To do this, we created Black-White morphed faces, which we labeled as either being Black or White. We predicted that, even when looking at the same target person, participants would assume that the target would feel less pain when the target was labeled “Black” vs. “White.”

### Methods

#### Participants

We recruited 99 participants via Mechanical Turk. We excluded 39 participants: 13 for not being native English-speakers and/or American and the rest for failing the manipulation checks. Including these participants does not change the results, however. The final sample of 60 varied in age (*M* = 30.98, *SD* = 11.24), gender (63% female), and race/ethnicity (73% White, 8% Black, 19% other).

#### Stimuli

We morphed a Black and a White male target face and a Black and a White female target face from Experiment 1 using FantaMorph software. The resulting male and female faces were racially ambiguous.

#### Procedure

The procedure was identical to that of Experiment 2, with the exception that all male participants saw the same (morphed) male target face and all female participants saw the same (morphed) female target face. Participants in the “Black target” condition were told that the racially-ambiguous target person was Black. Participants in the “White target” condition were told that the racially-ambiguous target person was White. After completing the pain ratings, participants were asked two questions about the target person: his/her name and his/her race/ethnicity.

### Results and Discussion

We constructed a GLM to examine the effect of target race on pain ratings, again controlling for participants’ age, gender, and self-ratings of pain. Analyses revealed a similar racial bias. Participants’ ratings were significantly lower for a Black vs. White target, *F*(1,53) = 5.97, *p* = .02, *d* = .67. See Figure 1. Because participants in both the Black target and White target conditions saw the same target faces, these differences cannot be attributed to differences in the target faces; they can only be attributed to the racial label ascribed to the target faces. In other words, the documented bias seems to be a race-related bias. Given the pervasiveness and potentially negative consequences of this bias, it is imperative to understand what is driving this effect. Experiments 5 and 6 begin to uncover the underlying mechanism of this bias.

## Experiment 5

In Experiment 5, we began to explore what psychological processes underlie this bias. Because this bias does not appear to be the direct result of racial prejudice (Experiment 1) or intergroup dynamics (Experiment 2), we looked to a social dimension beyond race; namely, status. We reasoned that the pain of lower-status individuals might be systematically underestimated because people assume that individuals who have had a life full of adversity are tough by necessity, whereas those who have had a life of privilege are frail by virtue of being sheltered and coddled. Because Blacks have relatively low status in U.S. society, people may assume that Black people have less privileged lives–lives with more hardships–and infer that they must be tougher. We tested this idea in Experiment 5 using a mediation approach.

### Methods

#### Participants

We recruited 127 participants via Mechanical Turk. Participants received $0.50 for their participation. We excluded 23 participants for not being native English-speakers and/or American. Including these participants in our analyses did not change the results below. For the sake of consistency across studies, however, we excluded these participants from the analyses. The final sample of 104 varied in age (*M* = 27.06, *SD* = 11.32), ethnicity (71% White, 9% Black, 20% other), and gender (51% female).

#### Procedure

Experiment 5 was a direct replication of Experiments 2 and 3 with one exception. At the end of the study, participants rated their own privilege on 4 items (i.e., how privileged do you think you are? How hard do you think your life has been? How lucky do you think you have been? How much adversity do you think you have overcome?) and the target person’s privilege using the same 4 items with anchors at 1-*Not at all* to 4-*Extremely*.

### Results and Discussion

We constructed a GLM to examine the effect of target race on perceptions of pain, controlling for age, gender, race, and self-ratings of pain. In replication of Experiments 1–4, participants’ pain ratings were lower for a Black vs. White target, *F*(1,98) = 3.67, *p* = .06, *d* = .39. See Figure 1.

We constructed a similar GLM to examine the effect of target race on perceptions of privilege, controlling for age, gender, race, and self-ratings of privilege. As expected, participants’ ratings of the target person’s privilege were significantly lower for a Black vs. White target, *F*(1,98) = 21.73, *p*<.0001, *d* = .94. In other words, participants assumed that the Black target was less privileged and faced more hardship than the White target.

**Figure 2:**
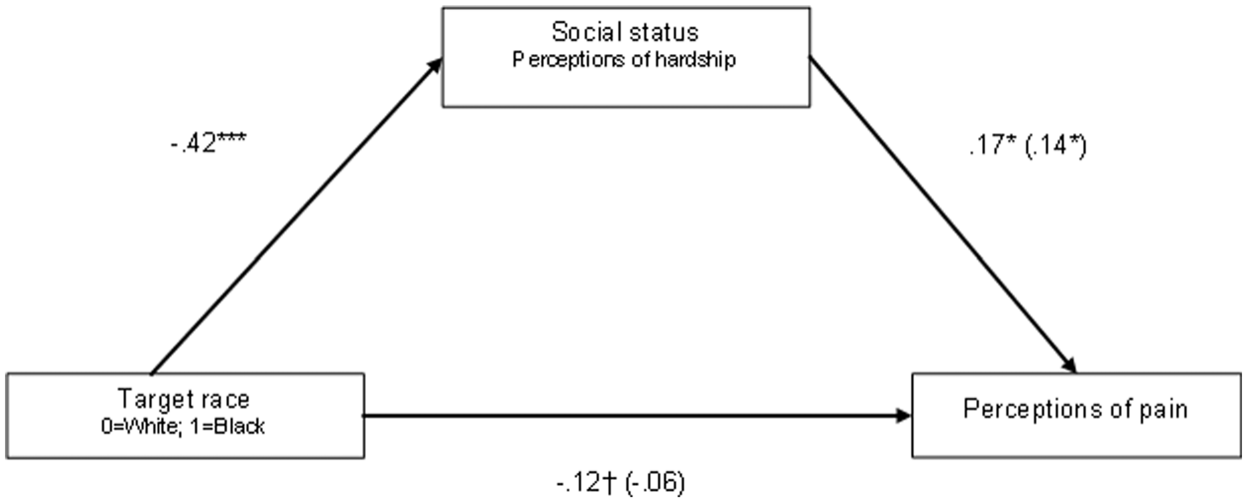
Mediation model for Experiment 5. All coefficients are standardized betas. Coefficients in parentheses are betas when controlling for perceptions of hardship. †*p* = .06; *p<.05; **p<.01; ***p<.001.

Finally, to examine whether perceptions of privilege mediated perceptions of pain, we regressed perceptions of pain onto perceptions of privilege, again controlling for all covariates. Consistent with predictions, participants’ ratings of the target’s privilege predicted pain ratings, *F*(1,97) = 7.39, *p* = .008, *d* = .55. The less privileged the target seemed, the less participants thought s/he would experience pain. In other words, participants associated hardship with physical toughness. Importantly, target race (Black vs. White) was no longer predictive of pain ratings once we controlled for participants’ perceptions of the target’s privilege, *F*<1, while target’s privilege continued to predict pain ratings *F*(1,96) = 3.98, *p* = .05, *d* = .41, Sobel test *z* = −2.42, *p* = .02. See Figure 2 for mediation model. These data suggest that perceptions of social status–how much privilege/hardship a person has experienced in life–mediate perceptions of pain. Perceived privilege/hardship accounted for the racial bias in perceptions of others’ pain.

## Experiment 6

In Experiment 6, we examined the effect of perceived privilege on perceptions of pain using a moderation approach and using a different operationalization of privilege. In particular, we tested whether giving participants information about the status of the target person might undo the racial bias. Specifically, we wanted to test whether participants would perceive a lower-status person as feeling significantly less pain than a higher-status person. If the bias we have documented is really about status and the privilege or hardship that status confers, as Experiment 5 suggests, then experimentally manipulating the target person’s status should moderate the racial bias.

### Methods

#### Participants

We recruited 302 participants via Mechanical Turk. Participants received $0.50 for their participation. We excluded 23 participants for not being native English-speakers and/or American and 34 for failing the manipulation checks (not remembering the target’s status or race, or giving the same answer for all questions in the study). Including these participants in our analyses changed the results below slightly–the pattern did not change but the difference between status conditions became marginally significant. The final sample of 245 varied in age (*M* = 31.73, *SD* = 11.71), ethnicity (84% White, 5% Black, 11% other), and gender (61% female).

#### Procedure

We used eight pictures of middle-aged, Black and White target persons in business attire. Participants were randomly assigned to view a gender-matched target. They were told to imagine that this person was of lower-, equal-, or higher-status, as a manipulation of perceived privilege. Specifically, participants in the lower-status condition were told, “Imagine that you and Jordan both work at the same company. He is your subordinate. You dictate and oversee his day-to-day tasks. He depends on your recommendation for promotions and salary increases.” Participants in the equal-status condition were told, “Imagine that you and Jordan are associates at the same company. You both have a manager who dictates and oversees your day-to-day tasks. You both depend on his recommendation for promotions and salary increases.” Participants in the higher-status condition were told, “Imagine that you and Jordan both work at the same company. He is your superior. He dictates and oversees your day-to-day tasks. You depend on his recommendation for promotions and salary increases.” We manipulated relative status rather than absolute status because status is relative–what is high status for one person may not be high status for another person. This manipulation complements the operationalization of privilege in Experiment 5, assessing perceived privilege through a closely related construct, social status [28]. Next, participants were asked to report how much pain they would feel if they accidentally stapled their own hand with an industrial stapler and how much pain this other person would feel if s/he accidentally stapled their hand with an industrial stapler. Participants made these ratings on 6-point scales (1-*not at all painful*, 6-*extremely painful*). Participants then answered questions about their perceptions of the target; namely, how similar they felt to the target person and how much control the target person ostensibly had over their outcomes. Again, they made these ratings on 6-point scales. Lastly, participants answered demographic questions and manipulation checks (e.g., questions about the status and race of the target).

### Results and Discussion

**Figure 3:**
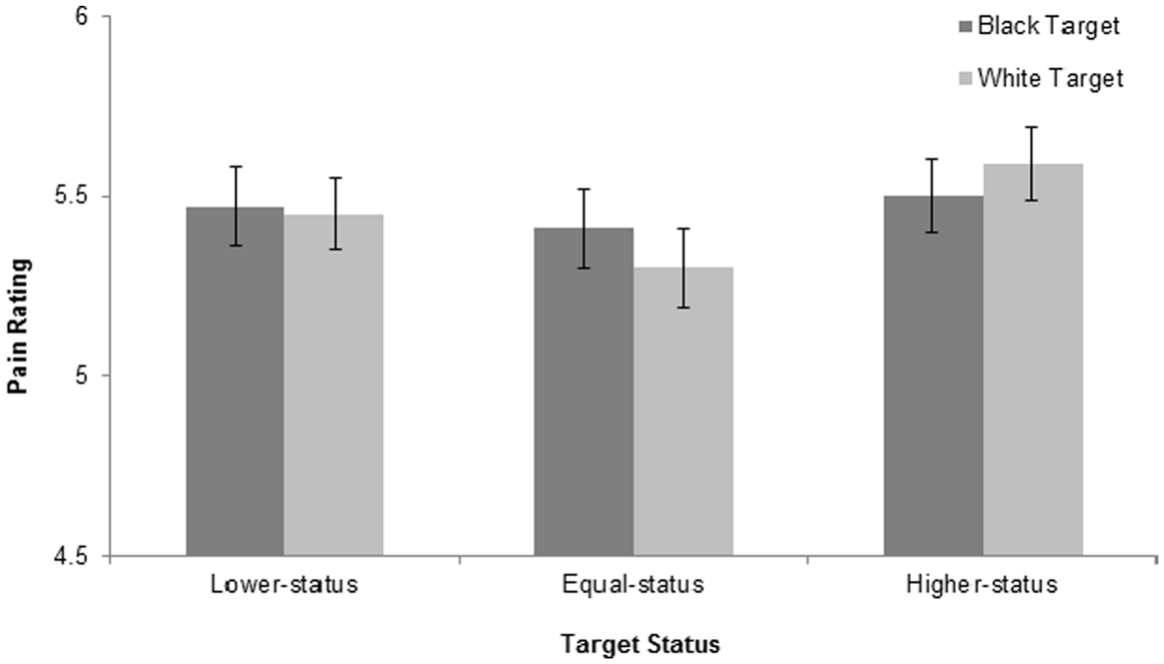
Pain ratings (estimated means and standard errors) for Experiment 6.

We constructed a GLM to examine the effect of target race, target status, and their interaction, controlling for age, gender, race, and self-ratings of pain. Results revealed that target status indeed affected participants’ ratings of the target’s pain, *F*(2,230) = 3.78, *p* = .02, η^2^ = .03. This effect was not moderated by target race, *F*<1. See Figure 3. Our a priori (linear) contrast comparing lower-, equal-, and higher-status targets (−1 0 1) was significant, *F*(1,230) = 5.91, *p* = .02, such that lower status resulted in lower pain ratings. As can be seen in Figure 3, however, the means of participants in the same-status condition are comparable to the means of participants in the lower-status condition. Although we predicted that the means of participants in the same-status condition would fall in-between the means of participants in the lower- and higher-status condition, we think that our same-status prompt may have conveyed low-status; i.e., “you both have a manager who dictates and oversees your day-to-day tasks. You both depend on his recommendation for promotions and salary increases” may have communicated to participants that the target person (and they themselves) had low status.

#### Secondary analyses

We also examined participants’ impressions of the target person. Analyses revealed that participants’ ratings of how much control the target ostensibly had over their outcomes predicted pain ratings, *F*(1,234) = 7.24, *p* = .007, controlling for self-ratings of pain, gender, age, and race. Participants attributed less pain to a target they perceived as having less status and power. Participants’ ratings of how similar they felt to the target did not predict pain ratings, *F*<1, suggesting that perceptions of status and power, but not similarity, influenced perceptions of the target’s pain. Taken together, data from Experiments 5 and 6 suggest that people use information (or assumptions) about status to estimate others’ pain. People seem to have a more general stereotype about low-status people; namely, that they are tough. What this means is that this bias may generalize to other low-status groups and that, as long as Black Americans are perceived to be low-status in our society, their capacity for pain is likely to be underestimated.

## General Discussion

The present work demonstrates that people assume *a priori* that Blacks feel less pain than do Whites. This finding has important implications for understanding and reducing racial bias. It sheds new light on well-documented racial biases. Consider, for instance, the finding that White Americans condone police brutality against Black men relative to White men [15]. Although it may be that some Whites (and non-Whites) condone police brutality against Black men because they condone harm against Black men, it may also be the case that at least some people condone police brutality against Black men because they assume that Black men feel less pain. They may perceive the same violent act as less injurious in the case of Black victims. As another example, consider the finding that Whites are not distressed at seeing harm inflicted upon Black (vs. White) people [18]. While it may be that some Whites do not care about Black people and their pain, it may also be the case that at least some Whites fail to realize that Black people feel as much pain as White people. Although still alarming, this explanation is decidedly different from the claim that White people simply do not care about Black people.

In the context of healthcare, our findings imply that one reason Black patients are undertreated for pain may be that medical personnel assume that Black patients feel less pain than do White patients. On the one hand, this is a more charitable attribution than blatant racism and the notion that (at least some) medical personnel withhold medication from Black patients. On the other hand, this bias may pose a more pernicious problem. Interventions aimed at reducing racial disparities in healthcare will need to target not only treatment but diagnosis of pain and illness. To that end, less subjective pain assessment methods need to be developed. In addition, simple mental exercises, such as perspective-taking, could be used. Research has shown that taking the perspective of patients can effectively reduce racial bias in pain treatment and improve Black patients’ satisfaction [5], [29], although our data suggest that such exercises will need to challenge assumptions about patients’ status to be effective. The present work also implies that current “paternalistic” models of doctor-patient relationships–whereby patients depend on their doctors but not vice versa–may unwittingly increase bias in perceptions of patients’ pain. In contrast, collaborative models, whereby doctors and patients depend on each other to reach mutually-satisfying outcomes, may reduce this bias. Future work should examine this possibility.

In sum, the present work finds that people assume that, relative to Whites, Blacks feel less pain because they have faced more hardship. At first blush, this assumption seems innocuous, even complimentary. It acknowledges the hardship Black people have faced and glorifies their strength and resilience. Nonetheless, this assumption leads to racial bias and potentially disastrous outcomes (e.g., condoning policy brutality against Blacks, underestimating and undertreating Black patients’ pain). Therein lies the problem.

## Supporting Information

Table S1 **Unadjusted means and standard deviations for self-ratings and ratings of others’ pain.**
(DOCX)

Table S2 **Zero-order correlations between self-ratings and ratings of others’ pain.**
(DOCX)
