# Correction: Racial Bias in Perceptions of Others' Pain

**DOI:** 10.1371/journal.pone.0152334

**Published:** 2016-03-24

**Authors:** Sophie Trawalter, Kelly M. Hoffman, Adam Waytz

There are errors in the Participants subsection of the Methods section of Experiment 2. The correct subsection is: We recruited 32 Black participants from the UVA Psychology pool (N = 12) and via Mechanical Turk (N = 20). UVA participants received course credit for their participation. Mechanical Turk participants received $0.50 for their participation. We excluded 5 participants from the primary analyses for not being native-English speakers and/or US-born. Including these participants in our analyses does not change the pattern of results. Our final sample of 27 varied in age (M = 31.74, SD = 14.18) and gender (70% female).

There are errors in the last two sentences of the Results and Discussion section of Experiment 2. The correct sentences are: Participants’ ratings were marginally lower for a Black vs. White target and sizeable, F (1, 22) = 3.01, p = .097, η_p^2 = .12. Of note, among the full sample of 32 participants, participants’ ratings were significantly lower for a Black vs. White target and even more sizeable, F (1, 27) = 7.89, p = .009, η_p^2 = .27.

There are errors in the Participants subsection of the Methods section of Experiment 3. The correct subsection is: We recruited 65 participants with the help of faculty members and administrators at a school of nursing. Participants were mailed a $10 gift certificate for their participation. We excluded 7 participants from the analyses for not being native-English speakers and/or US-born, and 14 more who identified the main hypothesis. It is worth noting that most of these participants completed the study toward the end of data collection, suggesting that they had heard about the study from someone else. Including these participants in our analyses did change the results—the pattern did not change but the difference between target race conditions was no longer statistically significant. The final sample of 43 included 29 registered nurses and 14 nursing students. The sample varied in age (M = 32.57, SD = 12.83) and ethnicity (88% White, 5% Black, and 7% other). All participants except one were women.

There is an error in the first sentence of the Participants subsection of the Methods section of Experiment 5. The correct sentence is: We recruited 127 participants via Mechanical Turk (N = 57) and via the UVA Psychology Department participant pool (N = 70).

There is an error in the last sentence of the Procedure subsection of the Methods section of Experiment 5. The correct sentence is: We averaged the privilege, hardship, and adversity items to form a privilege composite (=). A composite using all 4 items yields similar results, however.

There are errors in the second and third sentences of the Participants subsection of the Methods section of Experiment 6. The correct sentences are: We excluded 23 participants for not being native English-speakers and/or American and 34 for failing the manipulation checks (not remembering the target’s status or race, and one participant whose pain rating was more than 4 standard deviations below the grand mean). Including these participants in our analyses changed the results below—the difference between status conditions on pain ratings became non-significant; the difference between status conditions and perceptions of status/power remained highly significant and the relationship between perceptions of status/power and pain also remained highly significant.

There is an error in the fifth sentence of the Results and Discussion section of Experiment 6. The correct sentence is: Our a priori (linear) contrast comparing lower- to higher-status targets (-1 0 1) was not significant; however, a post-hoc contrast comparing the lower-status and equal-status target to the higher-status target (-1 -1 2) was significant, F (1, 230) = 5.91, p = .02.

There is an error in the fourth sentence of the Secondary Analyses subsection of the Results and Discussion section of Experiment 6. The correct sentence is: And, in fact, perceptions of the target’s power over their outcomes mediates the relationship between condition and perceptions of the target’s pain; a bootstrap analysis yields a 95% confidence interval that does not include 0, (95% CI [.0077, .094], p = .031).
