# Supplementary material for: Racial Bias in Perceptions of Others’ Pain
Source: PLoS One. 2012 Nov 14;7(11):e48546. doi: 10.1371/journal.pone.0048546 (PMC3498378; doi:10.1371/journal.pone.0048546)
Supplement: Table S1 — Unadjusted means and standard deviations for self-ratings and ratings of others’ pain. (DOCX) [file pone.0048546.s001.docx]

Table S1

| Condition | Target | Experiment 1 | Experiment 2 | Experiment 3 | Experiment 4 | Experiment 5 |
| --- | --- | --- | --- | --- | --- | --- |
| **White Target** | **Other** | **2.39**  **(.41)** | **2.60**  **(.51)** | **2.32**  **(.37)** | **2.39**  **(.42)** | **2.41**  **(.42)** |
|  | Self | 2.36  (.35) | 2.51  (.46) | 2.26  (.29) | 2.28  (.36) | 2.35  (.37) |
| **Black Target** | **Other** | **2.20**  **(.35)** | **2.46**  **(.48)** | **2.33**  **(.30)** | **2.34**  **(.45)** | **2.25**  **(.47)** |
|  | Self | 2.30  (.33) | 2.56  (.40) | 2.42  (.29) | 2.41  (.39) | 2.29  (.43) |

NOTE: Across studies, participants generally rated their own pain as more similar to a White (vs. Black) target, and greater than that of a Black (vs. White) target. This pattern is consistent with our interpretation that people underestimate or generally overlook Black people’s pain. There were exceptions in our data, however. In Experiment 3, for example, it appears that randomization to target race condition failed. The target race effect was driven by self-ratings (which, recall, participants made *before* seeing a Black or White target).
