# Supplementary material for: Racial Bias in Perceptions of Others’ Pain
Source: PLoS One. 2012 Nov 14;7(11):e48546. doi: 10.1371/journal.pone.0048546 (PMC3498378; doi:10.1371/journal.pone.0048546)
Supplement: Table S2 — Zero-order correlations between self-ratings and ratings of others’ pain. (DOCX) [file pone.0048546.s002.docx]

Table S2

| Condition | Experiment 1 | Experiment 2 | Experiment 3 | Experiment 4 | Experiment 5 |
| --- | --- | --- | --- | --- | --- |
| White Target | .50 | .81 | .73 | .62 | .77 |
| Black Target | .69 | .83 | .79 | .68 | .76 |

NOTE: Self-other correlations were comparable for White and Black targets, perhaps contrary to what one might expect (i.e., one might expect correlations to be larger between self and a White vs. Black target).
